# Comment on ‘A Causal Effect of Serum 25(OH)D Level on Appendicular Muscle Mass: Evidence From NHANES Data and Mendelian Randomization Analyses’ by Ren et al.

**DOI:** 10.1002/jcsm.70035

**Published:** 2025-07-25

**Authors:** Guanghao Zheng, Yu Cheng, Tao Zeng

**Affiliations:** ^1^ The Second Affiliated Hospital Jiangxi Medical College, Nanchang University Nanchang China; ^2^ Department of Urology The Second Affiliated Hospital of Nanchang University Nanchang China; ^3^ Department of Medicine, Jiangxi Medical College Nanchang University Nanchang China; ^4^ Nanchang University Nanchang China

Dear Editor,

We read with great interest the article titled ‘A Causal Effect of Serum 25(OH)D Level on Appendicular Muscle Mass: Evidence From NHANES Data and Mendelian Randomization Analyses’ in your esteemed journal [1]. By innovatively integrating cross‐sectional observational analysis with Mendelian Randomization (MR) approaches, this study provides important insights into the potential causal role of 25(OH)D status in appendicular muscle mass (AMM) maintenance, offering valuable implications for developing targeted interventions against sarcopenia, particularly in male populations. The authors established a rigorous analytical framework through two complementary phases: First, multivariable‐adjusted regression models based on NHANES data consistently demonstrated a dose‐dependent association between serum 25(OH)D concentrations and AMM. Building on these observational findings, the investigators performed two‐sample MR analyses using genome‐wide significant single nucleotide polymorphisms (SNPs) associated with 25(OH)D levels. The MR results revealed a positive causal relationship between genetically predicted 25(OH)D levels and AMM. This methodological progression from observational epidemiology to causal inference not only mitigates residual confounding inherent in observational designs but also provides compelling genetic evidence supporting 25(OH)D supplementation as a potential sex‐specific preventive strategy against sarcopenia, particularly advancing our understanding of musculoskeletal health in males. However, to enhance the rigour and reliability of the findings, we would like to offer several constructive suggestions regarding key aspects of the study design.

In MR analyses, instrumental variables (IVs) must satisfy three core assumptions: relevance, independence and exclusion restriction assumption [2]. While the authors have thoroughly discussed these assumptions and implemented stringent quality control measures, further scrutiny using the LDtrait database revealed that several selected SNPs (e.g., rs1047891, rs11076175, rs11204743, rs1260326, rs2756119, rs4616820, rs512083, rs55707527, rs55872725, rs6011153, rs72862854, rs7528419, rs7864910, rs804281 and rs8107974) are significantly associated with the outcome variable, AMM [3]. This suggests the potential presence of horizontal pleiotropy, which may violate the exclusion restriction assumption and compromise the accuracy of causal inference. To improve robustness, we recommend conducting additional forward and reverse MR analyses, excluding SNPs with possible pleiotropic effects.

Secondly, a key prerequisite of two‐sample MR is that the exposure and outcome data are derived from two independent, nonoverlapping populations with similar demographic characteristics [2]. Unfortunately, it appears that this has not been done by Ren et al. In this study, both the GWAS data for serum 25(OH)D levels and AMM were sourced from the UK Biobank, which raises the possibility of sample overlap bias. This can increase the risk of false positives due to weak instrument bias and the so‐called ‘winner's curse’ [4]. To address this, we recommend using publicly available non‐overlapping genetic datasets where feasible, and excluding case samples from case–control studies when estimating genetic associations with serum 25(OH)D, thereby enhancing the credibility of the causal estimates [4]. Additionally, although Ren and colleagues present the MR estimate of the effect of 25(OH)D on AMM as ‘Beta’, the corresponding units are not clearly defined. This lack of specification introduces ambiguity, particularly from a clinical interpretation standpoint. While ‘Beta’ is a standard term in linear regression, reporting the effect size with appropriate units would significantly improve the clarity and translational relevance of the findings, even if the statistical calculations are correct.

In the results section, the outcomes were stratified by sex (total, male and female population); however, the SNPs used as IVs for 25(OH)D levels were not sex‐specific. This discrepancy may introduce confounding or lead to misleading conclusions, as exposure and outcome variables may not align in terms of sex specificity. We recommend identifying sex‐specific SNPs or utilizing sex‐stratified GWAS summary statistics in future analyses to improve the precision and interpretability of results. A similar perspective has been highlighted in related studies by Shao et al. [5].

Finally, the statement, ‘However, the DXA screening was only performed in adults aged 8–59 years in NHANES 2011–2018,’ the age range ‘8–59 years’ appears to be a typographical error. Considering the context, it is likely intended to be ‘18–59 years.’ Although minor, such errors may cause confusion among readers, and we suggest correcting this detail in a future revision.

In conclusion, we sincerely commend the authors for their interdisciplinary efforts and valuable contributions. This study provides compelling preliminary evidence for a causal relationship between 25(OH)D and AMM. Our suggestions are intended to offer constructive insights for future research and to enhance the robustness and credibility of such analyses. We thank the journal for providing a platform for academic dialogue and look forward to seeing more high‐quality, thought‐provoking work in this area.

## Ethics Statement

The authors have nothing to report.

## Conflicts of Interest

The authors declare no conflicts of interest.

## Data Availability

As this study is based solely on previously published information and did not involve any new data generation or analysis, data sharing is not applicable.
